# The Impact of Complementary Feeding on Fecal Microbiota in Exclusively Breast-Fed Infants with Cystic Fibrosis (A Descriptive Study)

**DOI:** 10.3390/nu16234071

**Published:** 2024-11-27

**Authors:** Andrea Asensio-Grau, María Garriga, Saioa Vicente, Ana Andrés, Carmen Ribes-Koninckx, Joaquim Calvo-Lerma

**Affiliations:** 1 ALISOST Research Group, Department of Preventive Medicine, Public Health, Food Sciences, Toxicology and Legal Medicine, Faculty of Pharmacy and Food Sciences, University of Valencia, 46010 Valencia, Spain; andrea.asensio@uv.es; 2 NutriCura PDig Joint Research Unit UPV, La Fe Health Research Institute, 46026 Valencia, Spain; aandres@tal.upv.es; 3 Cystic Fibrosis Unit, University Hospital Ramón y Cajal, 28034 Madrid, Spain; maria.garriga@salud.madrid.org (M.G.); saioa.vicente@salud.madrid.org (S.V.); 4 Food UPV, Polytechnic University of Valencia, 46022 Valencia, Spain; 5 Celiac Disease and Digestive Immunopathology Research Group, Health Research Institute La Fe, 46026 Valencia, Spain

**Keywords:** breastfeeding, complementary feeding, cystic fibrosis, dysbiosis, gut microbiota, infants

## Abstract

Background/Objectives: Early life gut microbiota plays a pivotal role in shaping immunity, metabolism, and overall health outcomes. This is relevant in healthy infants but may be even more crucial in infants with chronic devastating diseases, such as cystic fibrosis (CF). While the introduction of solid foods in healthy infants modifies the composition of colonic microbiota, less knowledge is available on those with CF. The aim of this descriptive observational study was to assess the composition of fecal microbiota in six exclusively breast-fed infants with CF, and then explore the changes induced upon the introduction of different foods. Methods: two types of fecal samples were collected from each subject: one during the exclusive-breastfeeding period, and the other after incorporating each new food in the ad libitum diet. The microbiota composition was analyzed by 16S rRNA amplicon sequencing. Results: Wide heterogenicity in the composition at the phylum level (variable proportions of Actinobacteriota, Proteobacteria, and Firmicutes, and the absence of Bacteroidota in all subjects) was found, and different enterotypes were characterized in each subject by the main presence of one genus: *Bifidobacterium* in Subject 1 (relative abundance of 54.4%), *Klebsiella* in Subject 3 (49.1%), *Veillonella* in Subjects 4 and 5 (32.7% and 36.9%, respectively), and *Clostridium* in Subject 6 (48.9%). The transition to complementary feeding induced variable changes in microbiota composition, suggesting a subject-specific response and highlighting the importance of inter-individual variation. Conclusions: Further studies are required to identify which foods contribute to shaping colonic microbiota in the most favorable way for patients with CF using a personalized approach.

## 1. Introduction

Cystic fibrosis (CF) is an autosomal recessive disorder characterized by mutations in the cystic fibrosis transmembrane conductance regulator (CFTR) gene, leading to impaired chloride and sodium transport across epithelial cells. This disruption results in the production of thick, sticky mucus that primarily affects the lungs and digestive system, including the pancreas, liver, and intestine. Consequently, individuals with CF commonly experience malabsorption of nutrients, altered gut motility, and an increased risk of gastrointestinal infections, all of which contribute to dysbiosis or imbalances in the gut microbiota [1].

Early-life gut microbiota play a pivotal role in shaping immune development, metabolic processes, and overall health outcomes [2]. In healthy infants, breastfeeding is known to promote the establishment of a beneficial microbiota, rich in *Bifidobacterium* and *Lactobacillus* species, which protect against pathogens and promote gut integrity [3]. However, infants with CF may exhibit a distinct gut microbiota profile compared to healthy counterparts, characterized by a reduction in beneficial microbes and an increase in opportunistic pathogens [4]. This altered microbiota may further compromise their nutritional status and contribute to disease progression [5]. In this sense, the concept of cystic fibrosis-related gut microbiota (CFRGD) is currently used to refer to the specific reduced diversity and imbalance between proinflammatory and beneficial bacteria in this population group [6].

Complementary feeding is a crucial step in infant development, providing essential nutrients that may not be adequately supplied by breast milk alone. According to the current recommendations, complementary feeding should start no later than the age of 6 months, with foods being introduced gradually and as a support to breastfeeding [7]. The introduction of solid foods has been shown to influence the composition of the gut microbiota, promoting a shift from a milk-based to a more diverse diet-driven microbiome [8]. However, the impact of introducing complementary feeding in exclusively breast-fed infants with CF remains underexplored. Given the unique gastrointestinal circumstances associated with CF [9], understanding how complementary feeding modulates the fecal microbiota in these infants could provide insights into optimizing nutritional interventions to support gut health and disease management [10].

Previous studies have focused on comparing colonic microbiota before and after several weeks of enrolment in complementary feeding [11], but little information is available on the specific role of the type of food in modifying microbiota. Another recent study revealed that introducing complementary feeding could either help restore some balance or potentially exacerbate dysbiosis, depending on the timing and type of food introduced [12]. In the specific case of infants with CF, current studies on gut microbiota have focused on establishing possible functional alterations [13,14] rather than assessing the impact of solid foods on modulating the microbiota composition during the exclusive breastfeeding period.

Therefore, this descriptive study aims to investigate the changes in fecal microbiota composition following the introduction of complementary feeding in exclusively breast-fed infants with CF. By characterizing the shifts in microbial communities, we seek to identify subject-specific patterns that could contribute to the current knowledge on colonic microbiota in breast-fed infants with CF at the onset of complementary feeding.

## 2. Materials and Methods

### 2.1. Subjects and Study Design

A prospective, longitudinal, and observational study was conducted. It consisted of the collection of serial fecal samples from the moment before the introduction of complementary foods in exclusively breast-fed infants to after the introduction of different types of food. The introduction of foods was set ad libitum, with no common protocol for the subjects. The participants were advised to collect as many samples as possible within a period of 8 weeks from the moment of the introduction of the first food. The first sample belonged to the period of exclusive breast-feeding (t0), while the rest of the samples corresponded to the first stool after the introduction of a new food to the diet of the infant. The second stool was to be collected the day after the introduction of the new food: previous experimental studies showed significant changes in the microbiota composition and metabolism after 24 h of exposing basal colonic microbiota to a new food component or a prebiotic [15,16]. With this procedure, changes in the colonic microbiota could be attributed to the incorporation of a new food, minimizing the possible effects of other variables.

The inclusion criteria were an age younger than 6 months old, receiving exclusive breast-feeding, having a confirmed diagnosis of CF, and with the absence of antibiotic use during the last 2 weeks prior to inclusion. Infants using probiotics were excluded. During a regular visit to the CF Unit of University Hospital Ramón y Cajal (Madrid, Spain), the families of exclusively breast-fed infants with CF were invited to participate in this study. After signing informed consent, clinical data (age, gender, mutation, and mode of delivery) were retrieved from the medical records, and the infants’ height and weight were measured. The participants were instructed on the study protocol and given plastic containers to collect fecal samples. This study was approved by the Ethics Committees of University Hospital Ramón y Cajal (Madrid, Ref: HIPCI V1.0 04-04-2023 167/23) (approval date 1 June 2023) and Polytechnic University Hospital La Fe (Valencia, Ref. CEIm-F-PE-01-11 v01, 545/23) (approval date 4 May 2023). This study was conducted according to the standards of the Declaration of Helsinki.

### 2.2. Fecal Samples Collection and Analysis

The fecal samples were collected by the parents from the nappies of the study subjects immediately after deposition in a sterile plastic container. Then, the sample was marked with the code of the study subject, the ordinal number of the deposition, and the name of the food(s) that had been incorporated before the deposition. Then, the samples were kept in the household freezer at −20 °C. Once the 8-week period of the study was over, a carrier collected the samples from the houses of the participants and transported them on dry ice to the analytical laboratory (Microomics Systems S.L, Barcelona, Spain).

The composition and structure of the microbial communities was assessed through amplification and sequencing of the V3–V4 variable regions of the 16S rRNA gene. Briefly, amplification was performed after 25 PCR cycles. In this procedure, positive (CM) and negative (CN) controls were used to ensure quality control. The positive control was a mock community control, and it was processed in the same way as the samples. The obtained libraries were sequenced using Illumina Miseq (300 × 2). Raw demultiplexed forward and reverse reads were processed using QIIME2 [17]. DADA2 [18] is software that allows the following steps to be performed: (1) Read trimming. The 5′ adapters of reads were trimmed by the length of the used primer + 2 nt. (2) Quality filtering. The 3′ reads were trimmed to remove low-quality bases. (3) Denoising and pair-end merging. Forward and reverse reads were merged to rebuild amplicons. (4) Phylotype calling. Biological entities estimated on sequence data were called using the DADA2 pipeline. Quantification of bacteria at different taxonomic levels was expressed as the relative abundance (%)

### 2.3. Anthropometric Measurements

The infants’ height and weight were measured by the pediatrician, and the z-scores for height and weight were calculated according to the WHO 2006/2007 reference using the online SEGHNP Nutrition Application (www.seghnp.org/nutricional/ (accessed on 11 September 2024)) (Spanish Society for Pediatric Gastroenterology, Hepatology and Nutrition) [19].

### 2.4. Analysis of Alpha and Beta Diversity of Fecal Microbiota

Alpha diversity refers to the microbial diversity within a sample. Alpha diversity richness, also known as amplicon sequence variants (ASVs), is defined as the number of different phylotypes present in a community. Evenness is defined as the Pielou’s evenness index, which quantifies how equal the community is numerically; it considers the number and the abundance of phylotypes in a community. Phylotype data were used to calculate the alpha diversity metrics, and comparisons were performed using a generalized linear model with R package MASS v.7.3-54 [20].

Beta diversity describes differences in species composition among different samples or groups, i.e., it quantifies the variation in microbial composition in one sample compared to another. The Bray–Curtis index was selected to calculate the beta diversity. This method calculates the dissimilarity among bacterial communities, taking into account not only the number of different species but also the abundance. Distance matrices were used to calculate principal coordinates analysis (PCoA) and to make ordination plots using R software package version 4.2.0. The significance of groups was tested using Permanova and ANOSIM tests. Permdisp test was used to identify the location vs. dispersion effects [21]. The significance threshold was set at 0.05.

## 3. Results and Discussion

### 3.1. Subject and Sample Characteristics

A total of six exclusively breast-fed infants with CF were enrolled in this study, none of them having started complementary feeding at the time of the study onset (Table 1). Clinical management and follow-up were performed by the same health professionals in the CF team, which included a highly experienced pediatric gastroenterologist and a dietitian. Most of the subjects were females, and their age range was between 1.6 and 6.1 months old at the beginning of the study. Five of them had the F508del mutation in heterozygosis, and two were born by C-section. Except for subject 3, the participants had a nutritional status characterized by a z-score < 0 for height and weight. The fecal elastase levels, which are indicative of pancreatic function, were below 15 µg/g feces (exocrine pancreatic insufficiency in three subjects, while in two of them, the levels were >500, suggesting normal pancreatic function [22].

### 3.2. Basal Fecal Microbiota During the Exclusive Breastfeeding Period

**Figure 1:**
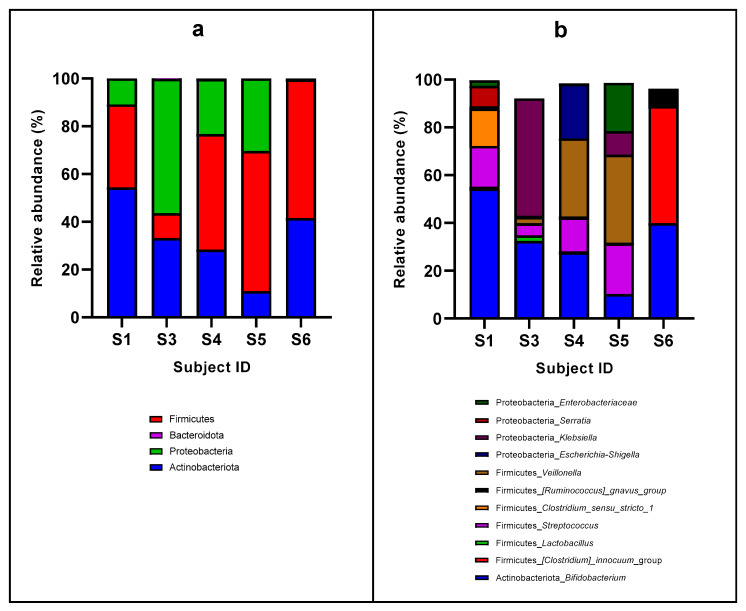
Baseline microbiota compositions in the study subjects at the phylum taxonomic level (**a**) and genus level (**b**).

Analysis of the fecal microbiota during the exclusive breastfeeding period was conducted in 5 out of 6 subjects, as subject 2 did not provide a fecal sample in this period (Figure 1). The results at the phylum level revealed that Firmicutes, Proteobacteria, and Actinobacteriota comprised >99% of the microbiota composition (Figure 1a). This finding aligns with previous studies indicating that these phyla are prominent in the gut microbiota of infants, particularly those undergoing exclusive breastfeeding [23,24]. However, a low abundance of Bacteroidota was present, with <1%, in subjects 3 and 4 and absent in the others. This reduced presence of Bacteroidota may reflect a deviation from the typical microbiota composition observed in healthy infants, where Bacteroidota are usually more abundant [25]. Notably, the absence of this phylum at such an early stage could have later implications in several outcomes [26]. The similarity in the enterotype between subjects 4 and 5, with Firmicutes as the predominant phylum (>50%), followed by Proteobacteria and Actinobacteriota, indicates a convergent microbial profile that may be influenced by shared factors, such as breastmilk composition or environmental exposures. These subjects presented with similar clinical characteristics in terms of pancreatic insufficiency (fecal elastase), nutritional status indicators, age, and pancreatic insufficiency-related mutations. Subject 1 also exhibited a similar profile, although with a higher proportion of Actinobacteriota compared to Firmicutes. In turn, in subject 3, the microbiota were notably dominated by Proteobacteria (>50%), with Actinobacteriota and a small proportion of Firmicutes. This predominance of Proteobacteria is particularly interesting, as high levels have been associated with dysbiosis and inflammation in various contexts [27]. The imbalance in microbial composition could potentially contribute to the inflammatory and gastrointestinal symptoms commonly observed in CF. However, contrasting with this finding, subject 3 had indicators of the best nutritional status of all the series and fecal elastase levels, suggesting a lack of digestive affection. Therefore, this finding supports the multifactorial origin of microbiota composition [6]. Finally, subject 6 was characterized by only two phyla, namely Firmicutes and Actinobacteriota. This unique profile might reflect an atypical microbial colonization pattern, which could be related to the individual’s specific health status or management of CF.

Detailed analysis of the fecal microbiota at the genus level during the exclusive breastfeeding period revealed distinct enterotypes, each characterized by a dominant genus (Figure 1b). This granularity in microbial composition offers additional insights into the microbial landscape and potential functional implications for these infants. In subject 1, *Bifidobacterium* was the predominant constituent of the fecal microbiota (relative abundance of 54.4%), and in subject 3, it was *Klebsiella* (49.1%). In subjects 4 and 5, *Veillonella* represented the most abundant genus (32.7% and 36.9%, respectively) and in subject 6, the *Clostridium innocuum* group was the predominant genus (48.9%). Common patterns could be found, as in all of them, *Bifidobacterium* was present in relatively high proportions (54.4–27.7%), except for subject 5, in which this genus accounted for a relative abundance of 10.3%. Also, *Streptococcus* was found in all the subjects (ranging from 21.1% to 0.58%). As for other specific results, the genus *Enterobacteriaceae* spp. was present in subjects 1 and 5 (2.3 and 20.2%, respectively), *Escherichia-Shigella* was only present in subject 4 (22.8%), Serratia in subject 6 (8.6%), and *Clostridium* sensu stricto in subject 1 (15.7%), although it was also present in the rest of the subjects in minor proportions (<0.5%). *Lactobacillus* was present in low proportions in subjects 1 and 4 (0.85% and 0.18%, respectively) and in subject 3 (2.34%).

The presence of *Bifidobacterium* in a relevant proportion aligns with the common finding that *Bifidobacterium* is a major component of infant gut microbiota, particularly during the breastfeeding period [28]. *Bifidobacterium* plays a crucial role in fermenting oligosaccharides from breast milk and contributing to gut health by producing beneficial metabolites [29]. Similarly, *Streptococcus* was detected in all subjects, although its abundance varied notably from 21.1% to 0.58%. *Streptococcus* is a common member of oral and gut microbiota and can reflect transitional microbial states or varying environmental exposures [30]. In contrast, in subject 3, the microbiota were dominated by *Klebsiella* (49.1%), a genus typically associated with pathogenic potential, especially in the context of dysbiosis [31]. The high relative abundance of *Klebsiella* in this subject suggests an imbalance in microbial homeostasis or an adaptive response to the specific conditions of CF. The high levels of *Veillonella* in subjects 4 and 5 indicate a microbiota profile adapted to the high-lactate environment in the gut, potentially influenced by breastfeeding or the underlying condition of CF [32]. The high proportion of *Veillonella* in these subjects contrasts with the lower levels found in others, suggesting significant variability in microbial composition among infants with CF. Moreover, in subject 6, the domination of the *Clostridium innocuum* group (partly explaining the high proportion of Proteobacteria) can be an indicator of dysbiosis disrupting the normal microbial community [33].

Analysis of the microbiota at the phylum and genus taxonomic levels during the exclusive breastfeeding period revealed variation in the compositions, underscoring the heterogeneity of microbial communities even with a specific condition like CF. The results coincide with the current knowledge on microbiota in children with CF [34], which can be further influenced by individual host factors, differences in breastfeeding practices, breastmilk composition [35], and interactions among bacterial groups [36].

### 3.3. Changes in Fecal Microbiota Composition upon Introduction of Complementary Feeding

The longitudinal evaluation of changes in the basal fecal microbiota after starting complementary feeding was enabled in subjects 1, 2, and 3, who followed different food introduction patterns when introducing foods to their diets (Table 2). Subjects 4–6 dropped out because of the impossibility of complying with the study protocol.

**Figure 2:**
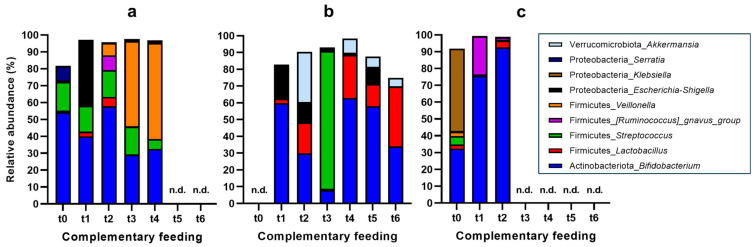
Evolution of microbiota composition over the complementary feeding follow-up period in study subjects 1 (**a**), 2 (**b**), and 3 (**c**), expressed as the relative abundance (%) at the genus taxonomic level. n.d., non-determined.

In terms of microbiota composition, the evolution over the introduction of complementary feeding upon finishing the breastfeeding period was different in each subject (Figure 2). Our findings suggest a subject-specific response of the microbiota to the introduction of foods and highlight the importance of considering inter-individual variation in microbiota studies.

Focusing first on subject 1 (Figure 2a), their *Bifidobacterium* levels decreased (−10%) after introducing infant formula at t1, but they returned to similar values as during the exclusive breastfeeding 24 h after the introduction of cereals to the diet, and then decreased to values close to 25–30% at the end of the follow-up period. The initial decrease could be related with the different nutrient and prebiotic profile of formulas compared to breastmilk [37]. The later increase could reflect a transitional adjustment to the new feeding regime, followed by a sustained modification in gut microbiota composition. A relevant finding was that the phylum *Escherichia-Shigella* debuted at t1 as a main constituent of the microbiota (35%), but it resulted in a minor proportion during the rest of the study period. Possibly, this change could be also attributed to the transition to an infant formula, which previously has been related with the proliferation of this genus [38]. Then, new dietary changes could have favored the growth of other bacterial groups, to the detriment of *Escherichia-Shigella*. Similarly, the *Ruminococcus gnavus* group was only present at t2 when a solid-fiber-containing food (cereal) was first introduced to the diet; this genus has the capability of adapting to the digestion of complex dietary components of complementary feeding [39]. *Streptococcus* was maintained at similar proportions during complementary feeding (15%) [8], except in the last time point, when it was reduced to <5%, possibly because of an adjustment in the colonic microbiota after dietary changes that could have enabled the growth of other bacterial groups at the expense of this genus. However, the most notable change was related to *Veillonella*, which appeared at t2 (7.21%), but largely increased to >50% at t3 (the introduction of fruit and cereal) and t4 (the introduction of chicken and vegetables). This increase suggests that the inclusion of a wider variety of foods could have benefited *Veillonella*’s ability to ferment the dietary sources introduced in this phase [40]. Of note, the initial appearance of *Escherichia-Shigella* at t1 and its subsequent reduction, along with the notable increase in *Veillonella* during later time points, suggests a dynamic interaction between these two microbial species in response to specific dietary components [41].

Regarding subject 2 (Figure 2b), they lacked a baseline breastfeeding fecal sample, limiting a direct comparison of the changes in their fecal microbiota. Following the evolution of *Bifidobacterium*, it started at t1 (after introducing fruit), with a relative abundance of 60%, being the predominant genus. This finding aligns with the known association of *Bifidobacterium* with a healthy gut microbiota in infants, especially in the context of a fruit-rich diet [42]. Then, it decreased (−30%) at t2 (the introduction of vegetables) and again (−20%) at t3 when chicken was incorporated into the diet. Despite these reductions, the levels of *Bifidobacterium* rebounded at t4 (the introduction of cereal), suggesting a resilient or adaptive response to dietary changes. However, the decline to 35% at t6 with the introduction of fish might indicate that *Bifidobacterium* struggles to compete with other taxa in certain dietary conditions [43]. The genus *Escherichia-Shigella* also showed variations over the follow-up period, as it started at a relative abundance of 19.2% (t1) and continued at 11.8% in t2, but it remained at <1% over the rest of the series, except in t5, when it raised to 9.2%. It was remarkable that when chicken was introduced (t3), *Streptococcus* (which was at <1% at the rest of the timepoints) dominated the composition, with a relative abundance of 82.1%. *Streptococcus* is known to be resilient in various environments, and its predominance during the introduction of chicken suggests a potential competitive advantage or adaptation to the new dietary components [44]. Additionally, there were two genera that were only observed in subject 2 (*Akkermansia*) or at a relative abundance of >5% (*Lactobacillus*). *Akkermansia* debuted at t2 (vegetables), with a relative abundance of 29.9%, and then it was reduced to <1% (t3), but recovered higher values that gradually decreased (8.32%–5.01%, t4–t6). In the case of *Lactobacillus*, an overall increasing tendency was depicted; although at some points it was low (1–2%), it represented a relative abundance of 18.3% at t2, 25.4% at t4, and 35.7% at t6.

Finally, only fruit and vegetables were introduced to the diet of subject 3 (Figure 2c) during the assessment period. Samples were collected from this subject at only two timepoints, presenting a clear evolution pattern of increasing *Bifidobacterium*, from 31% at baseline to 75.7% and 92.6% at t1 (introduction of fruit) and t2 (introduction of vegetables), respectively. The high increase in this genus was at the expense of the abrupt loss of the other predominant genera at baseline (49.1%), i.e., *Klebsiella*. Other observed changes were related to the 22.7% presence of the *Ruminococcus gnavus* group in t1 (reduced to 1% in t2) and the reduction of the initial *Veillonella* (2.58%) to 0.33% at t2. The significant increase in *Bifidobacterium* following the introduction of fruits and vegetables, accompanied by the reductions in *Klebsiella* and *Veillonella*, indicates a shift toward a microbiota profile associated with the consumption of fiber-rich foods. This shift is consistent with previous findings that fiber intake promotes the growth of beneficial bacteria, such as *Bifidobacterium* [45].

### 3.4. Microbiota Diversity from Exclusive Breastfeeding to Complementary Feeding

**Figure 3:**
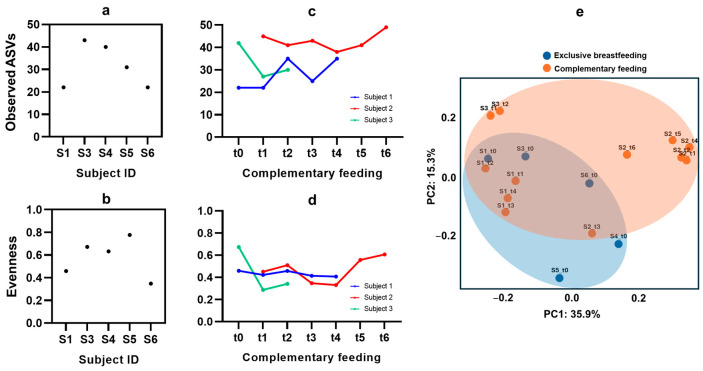
Fecal microbiota alpha diversity expressed as richness (observed ASVs) and evenness during the breastfeeding period ((**a**) and (**b**), respectively) and over the complementary feeding period ((**c**) and (**d**), respectively). Beta diversity (Bray–Curtis) expressed with PCoA and two groups of samples (breastfeeding and complementary feeding) (**e**).

Complementary to the follow-up of changes in microbiota composition, changes in the diversity were also assessed. As for the baseline microbiota, the richness (observed ASVs), which represents the number of different bacterial species, ranged from 22 to 43 (Figure 3a). These relatively low values are typically found in exclusively breastfed infants due a bacterial genus (*Bifidobacterium* in the general population) that dominates the composition, leaving fewer opportunities for other genera [46]. The low values are also comparable with the range of alpha diversity found in healthy breastfed infants, namely between 10 and 35 species during the first 3 months of life [47]. In terms of evenness (Figure 3b), the results are also coherent with the lower values found in breastfed infants compared to older children; for example, Bokulich et al. (2016) found values around of 0.3–0.5 during the early infancy of breastfed infants, increasing after the introduction of complementary foods [48]. Upon the introduction of solid foods, evenness can increase up to 0.6–0.8, as the gut microbiota become more balanced with the inclusion of a wider variety of substrates for microbial growth [49], but in infants with CF, one of the few available studies reported values ranging from 15 to 45 species, which aligns with the range found in our study [50] (Figure 3c).

Analysis of the beta diversity (Bray–Curtis) was conducted to assess how the incorporation of complementary feeding influenced the variability in the microbiota composition. In Figure 3e (PCoA), the beta diversity is visualized in terms of the distances between the different samples. The individual points (belonging to each fecal sample) that are represented together indicate similarity to each other. Focusing first on the subjects, the figure displays the separated aggrupation of the samples belonging to the same subject; for example, subject 2 (S2) is differentiated from the rest in the upper right side of the graph, except for the sample from t3 (the introduction of chicken, which led to an abrupt increase in the genus Streptococcus), which is represented in a different area. Focusing on subject 1, the samples belonging to the complementary feeding period (t1, t3, and t4) were found apart from the baseline sample belonging to the exclusive breastfeeding period (t0). For subject 3, in turn, a large distance is shown between the t0 sample and those from t1 and t2, which is consistent with the observed change in composition related to the increase in *Bifidobacterium* from t0 to t1, and the similar composition between t1 and t2. As for the rest of the subjects, only the samples from the exclusive breastfeeding period were available.

Taking into account the samples in two groups in terms of breastfeeding vs. complementary feeding, those from the second group showed more dispersion, suggesting that the samples from this group had more variability in their microbiota composition. In turn, samples from the exclusive breastfeeding period were more concentrated, indicating less variability among the samples. However, the PERMANOVA analysis that was applied to assess the dissimilarity between these two groups of samples revealed no statistically significant differences. The low number of subjects and the absence of samples from the complementary feeding period in three subjects may have prevented us from obtaining a more comprehensive assessment. According to current knowledge, both alpha and beta diversity tend to increase over complementary feeding compared to the lactation period [51]. Our sample size and follow-up time may have prevented us from observing a clear tendency toward increased diversity, as previous studies have led to this conclusion after longer assessment periods between 6 months and 2 years [23,49]. However, our study specifically aimed at assessing concrete changes in the microbiota composition and diversity during the weeks of transition from one feeding regime (exclusive breastfeeding) to another.

### 3.5. General Discussion

This study’s results emphasize the need for personalized approaches in complementary feeding and microbiota monitoring, considering individual responses to optimize health benefits in infants with CF. Overall, to explain the sole effect of food introduction on modifying gut microbiota, controlled in vitro digestion studies should be designed, as previously carried out by Asensio-Grau et al. (2024) [16]. In the context of in vitro-simulated digestion, individual factors other than the microbiota composition per se are omitted (transit time, degree of pancreatic insufficiency, appetite, etc.). Therefore, using a fecal microbiota extract from exclusively breastfed infants with CF to conduct the experiment, the changes after exposing microbiota to different foods could be exclusively attributed to the food characteristics in combination with the individual microbiota of each subject. Additionally, and despite the difficulty, future studies should address an intervention approach with more participants, all of them following a fixed pattern for introducing complementary feeding with common timing. This approach would enable exploring association or correlation statistical analysis between the subjects’ characteristics and their fecal microbiota composition, thus offering more concrete conclusions.

Our results can be compared with those of previous studies assessing the impact of complementary feeding on fecal microbiota in breast-fed infants with CF. Eng et al. (2021) also found altered microbiota (reduced diversity and the presence of specific genera) in their cohort of patients, attributed to specific disease characteristics, such as reduced nutrient absorption [13]. The authors highlighted that those CF-related microbiota patterns could have an impact on how nutrients from foods are fermented throughout colonic fermentation, while in our study, metabolite production was not assessed. Another in vitro digestion study by our group assessed the impact of introducing complementary feeding foods to the fecal microbiota of an exclusively breastfed infant with CF compared to a healthy infant [16]. The results showed that introducing foods differently affected the microbiota of the CF and healthy infants, causing a sharper decrease in *Bifidobacterium* in the infants with CF in favor of potentially pathogenic genera, such as *Veillonella* and *Escherichia-Shigella*. The results of the present study coincide with this pattern after the introduction of some specific foods in different subjects. However, the comparison in the previous study was made between two subjects, and the differences could be attributed to inter-subject variability and not so much to CF vs. non-CF conditions, as, in the present study, large variability among the subjects was also observed. Overall, the mentioned studies coincide with the message of this study: diet can play a crucial role in modulating microbiota composition in children with CF, and personalized complementary feeding approaches could be beneficial for patients.

Finally, the authors are aware of the limitations of this study. First, the sample size, due to the number of breast-fed infants with CF younger than 6 months old in Spain, was extremely reduced. Other hospitals with reference CF units could not participate in the study due to the lack of subjects meeting the inclusion criteria (<6 months old). Another limitation was the lack of a common pattern for introducing foods, which have prevented paired comparisons among the study timepoints. A proper study design would have included controlling for food intake, but this was discarded to avoid the risk of subjects not enrolling due to the additional burden of the treatments, so an ad libitum diet was allowed instead. In addition, from the three subjects that completed the collection during complementary feeding, one of them lacked a baseline fecal sample from the exclusive breastfeeding period. These limitations prevented us from conducting inferential statistical analyses, and the results are restricted to descriptive observations. However, the value of our research relies on the specific evaluation of microbiota composition changes in the transition from exclusive breastfeeding to complementary feeding. Also, we consider that assessing fecal microbiota composition in exclusively breast-fed infants with CF contributes to expanding scientific knowledge.

Overall, this study provides new insights into the diversity and composition of the faecal microbiota in infants with CF who are exclusively breastfed and during the transition to complementary feeding in a short series. During the exclusive breastfeeding period, significant interindividual variability was observed in the predominant gut microbiota genera, which aligns with previous research highlighting the influence of genetic, environmental, and breastfeeding factors in the establishment of the gut microbiota during the first months of life. In addition, the results show that the transition to the introduction of solid foods differently affect the microbiota composition depending on the subject, underscoring the complex interactions between diet and gut microbiota in infants with CF and highlighting the need for personalized approaches to dietary management in this population.

## 4. Conclusions

In conclusion, this study suggests that introducing complementary feeding in lactating infants with CF can be a relevant strategy to modulate colonic microbiota composition. However, the individual underlying dysbiosis already present at a very early stage, further conditioning the impact of the introduction of complementary feeding, remains a challenge. More specific studies are required to establish the specific effect of individual foods on modifying the microbiota of exclusively breast-fed infants with CF and to identify which foods contribute to shaping the colonic microbiota after the termination of exclusive breastfeeding in the most favorable way for patients with CF using a personalized approach.

## Figures and Tables

**Table 1:** Subject characteristics at study onset.

Subject	Age (Months)	Sex	Mutation 1	Mutation 2	Mode of Delivery	Fecal Elastase (µg/g Feces)	Weight (kg)	Weight (z-Score)	Height (cm)	Height (z-Score)
Subject 1	2.9	Female	F508del	N1303K	C-section	<15	4.81	−1.56	58.5	−0.65
Subject 2	6.1	Male	F508del	L206W	Vaginal	377	6.49	−1.83	66	−0.79
Subject 3	5.4	Female	F508del	3272-26A->G	C-section	>500	7.53	0.44	66	0.42
Subject 4	1.7	Female	F508del	L571S	Vaginal	<15	4.77	−0.18	55.5	−0.33
Subject 5	2.4	Female	1609delCA	2594delGT	Vaginal	<15	5.21	−0.4	56.5	−0.92
Subject 6	1.6	Female	F508del	L206W	Vaginal	>500	4.01	−1.34	53	−1.42

**Table 2:** Timing and foods incorporated during the complementary feeding period in the study subjects.

Subject	Time 0 (t0)	→	Time 1 (t1)	→	Time 2 (t2)	→	Time 3 (t3)	→	Time 4 (t4)	→	Time 5 (t5)	→	Time 6 (t6)
Subject 1	Exclusive breast-feeding	90 d	Infant formula	16 d	Cereal	10 d	Fruit + cereal	27 d	Chicken + vegetables		-		-
Subject 2	-		Fruit	4 d	Vegetables	4 d	Chicken	19 d	Cereals	13 d	Beef	16 d	Fish
Subject 3	Exclusive breast-feeding	18 d	Fruit	18 d	Vegetables		-		-		-		-

## Data Availability

Data will be made available upon reasonable request.

## References

[1] O’Sullivan B.P., Freedman S.D. (2009). Cystic fibrosis. Lancet.

[2] Arrieta M.-C., Stiemsma L.T., Amenyogbe N., Brown E.M., Finlay B. (2014). The intestinal microbiome in early life: Health and disease. Front. Immunol..

[3] Martín R., Miquel S., Ulmer J., Kechaou N., Langella P., Bermúdez-Humarán L.G. (2016). Role of commensal and probiotic bacteria in human health: A focus on inflammatory bowel disease. Microb. Cell Fact..

[4] Nielsen S., Needham B., Leach S.T., Day A.S., Jaffe A., Thomas T., Ooi C.Y. (2016). The gut microbiome of healthy and cystic fibrosis children. Microbiome.

[5] Enaud R., Prevel R., Ciarlo E., Beaufils F., Wieërs G., Guery B., Delhaes L. (2020). The gut-lung axis in health and respiratory diseases: A place for inter-organ and inter-kingdom crosstalks. Front. Cell. Infect. Microbiol..

[6] Caley L.R., White H., de Goffau M.C., Floto R.A., Parkhill J., Marsland B., Peckham D.G. (2023). Cystic fibrosis-related gut dysbiosis: A systematic review. Dig. Dis. Sci..

[7] World Health Organization (2023). WHO Guideline for Complementary Feeding of Infants and Young Children 6–23 Months of Age.

[8] Shi Y., Yin R., Pang J., Chen Y., Li Z., Su S., Wen Y. (2024). Impact of complementary feeding on infant gut microbiome, metabolites and early development. Food Funct..

[9] Gelfond D., Borowitz D. (2013). Gastrointestinal complications of cystic fibrosis. Clin. Gastroenterol. Hepatol..

[10] De Weerth C. (2017). Do bacteria shape our development? The gut microbiome and its role in health and disease during infancy. Front. Pediatr..

[11] Fallani M., Amarri S., Uusijarvi A., Adam R., Khanna S., Aguilera M., Gil A., Vieites J.M., Norin E., Young D. (2011). Determinants of the human infant intestinal microbiota after the introduction of first complementary foods in infant samples from five European centres. Microbiology.

[12] Differding M.K., Benjamin-Neelon S.E., Hoyo C., Østbye T., Mueller N.T. (2020). Timing of complementary feeding is associated with gut microbiota diversity and composition and short chain fatty acid concentrations over the first year of life. BMC Microbiol..

[13] Eng A., Hayden H.S., Pope C.E., Brittnacher M.J., Vo A.T., Weiss E.J., Hager K.R., Leung D.H., Heltshe S.L., Raftery D. (2021). Infants with cystic fibrosis have altered fecal functional capacities with potential clinical and metabolic consequences. BMC Microbiol..

[14] Thavamani A., Salem I., Sferra T.J., Sankararaman S. (2021). Impact of altered gut microbiota and its metabolites in cystic fibrosis. Metabolites.

[15] Asensio-Grau A., Heredia A., García-Hernández J., Cabrera-Rubio R., Masip E., Ribes-Koninckx C., Collado M.C., Andrés A., Calvo-Lerma J. (2024). Effect of beta-glucan supplementation on cystic fibrosis colonic microbiota: An in vitro study. Pediatr. Res..

[16] Asensio-Grau A., Calvo-Lerma J., Ribes-Koninckx C., Heredia A., Andrés A. (2024). Complementary feeding in infants with cystic fibrosis: In vitro nutrient digestibility and impact on colonic microbiota. Food Biosci..

[17] Bolyen E., Rideout J.R., Dillon M.R., Bokulich N.A., Abnet C.C., Al-Ghalith G.A., Alexander H., Alm E.J., Arumugam M., Asnicar F. (2019). Reproducible, interactive, scalable and extensible microbiome data science using QIIME 2. Nat. Biotechnol..

[18] Callahan B.J., Mcmurdie P.J., Rosen M.J., Han A.W., Johnson A.J.A., Holmes S.P. (2016). Dada2: High-resolution sample inference from Illumina amplicon data. Nat. Methods.

[19] Nutritional Application of the Spanish Society of Pediatric Gastroenterology, Hepatology and Nutrition (SEGHNP). https://www.seghnp.org/nutricional/.

[20] Venables W.N., Ripley B.D. (2002). Modern Applied Statistics with S.

[21] Anderson M.J., Walsh D.C.I. (2013). PERMANOVA, ANOSIM, and the Mantel test in the face of heterogeneous dispersions: What null hypothesis are you testing?. Ecol. Monogr..

[22] Daftary A., Acton J., Heubi J., Amin R. (2006). Fecal elastase-1: Utility in pancreatic function in cystic fibrosis. J. Cyst. Fibros..

[23] Yatsunenko T., Rey F.E., Manary M.J., Trehan I., Dominguez-Bello M.G., Contreras M., Magris M., Hidalgo G., Baldassano R.N., Anokhin A.P. (2012). Human gut microbiome viewed across age and geography. Nature.

[24] Kuang Y.S., Li S.H., Guo Y., Lu J.H., He J.R., Luo B.J., Qiu X. (2016). Composition of gut microbiota in infants in China and global comparison. Sci. Rep..

[25] Chen X., Yan Z., Liu L., Zhang R., Zhang X., Peng C., Hou X. (2022). Characteristics of gut microbiota of term small gestational age infants within 1 week and their relationship with neurodevelopment at 6 months. Front. Microbiol..

[26] Tzeng Y.-L., Jang M.-H. (2019). Development of gut microbiota in infants and its impact on early life health. Nutrients.

[27] Koren O., Knights D., Gonzalez A., Waldron L., Segata N., Knight R., Huttenhower C., Ley R. (2014). A guide to enterotypes across the human body: Meta-analysis of 16S rRNA microbial community data. Sci. Rep..

[28] Huda M.N., Lewis Z., Kalanetra K.M., Rashid M., Ahmad S.M., Raqib R., Qadri F., Underwood M.A., Mills D.A., Stephensen C.B. (2014). Stool microbiota and vaccine responses in infants. J. Pediatr..

[29] Moubareck C.A. (2021). Human milk microbiota and oligosaccharides: A glimpse into benefits, diversity, and correlations. Nutrients.

[30] Pantoja-Feliciano I.G., Clemente J.C., Costello E.K., Perez M.E., Blaser M.J., Knight R., Dominguez-Bello M.G. (2013). Biphasic assembly of the murine intestinal microbiota during early development. ISME J..

[31] Calderon-Gonzalez R., Lee A., Lopez-Campos G., Hancock S.J., Sa-Pessoa J., Dumigan A., McMullan R., Campbell E.L., Bengoechea J.A. (2023). Modelling the gastrointestinal carriage of Klebsiella pneumoniae infections. Mbio.

[32] Łoniewski I., Skonieczna-Żydecka K., Stachowska L., Fraszczyk-Tousty M., Tousty P., Łoniewska B. (2022). Breastfeeding affects concentration of faecal short chain fatty acids during the first year of life: Results of the systematic review and meta-analysis. Front. Nutr..

[33] Cherny K.E., Muscat E.B., Reyna M.E., Kociolek L.K. (2021). Clostridium innocuum: Microbiological and Clinical Characteristics of a Potential Emerging Pathogen. Anaerobe.

[34] Coffey M.J., Nielsen S., Wemheuer B., Kaakoush N.O., Garg M., Needham B., Pickford R., Jaffe A., Thomas T., Ooi C.Y. (2019). Gut microbiota in children with cystic fibrosis: A taxonomic and functional dysbiosis. Sci. Rep..

[35] Catassi G., Aloi M., Giorgio V., Gasbarrini A., Cammarota G., Ianiro G. (2024). The role of diet and nutritional interventions for the infant gut microbiome. Nutrients.

[36] Dethlefsen L., Relman D.A. (2011). Incomplete recovery and individualized responses of the human gut microbiota to repeated antibiotic perturbation. Proc. Natl. Acad. Sci. USA.

[37] Rinne M.M., Gueimonde M., Kalliomäki M., Hoppu U., Salminen S.J., Isolauri E. (2005). Similar bifidogenic effects of prebiotic-supplemented partially hydrolyzed infant formula and breastfeeding on infant gut microbiota. FEMS Microbiol. Immunol..

[38] Penders J., Vink C., Driessen C., London N., Thijs C., Stobberingh E.E. (2005). Quantification of Bifidobacterium spp., Escherichia coli and Clostridium difficile in faecal samples of breast-fed and formula-fed infants by real-time PCR. FEMS Microbiol. Lett..

[39] Crost E.H., Le Gall G., Laverde-Gomez J.A., Mukhopadhya I., Flint H.J., Juge N. (2018). Mechanistic insights into the cross-feeding of Ruminococcus gnavus and Ruminococcus bromii on host and dietary carbohydrates. Front. Microbiol..

[40] den Bogert B.V., Erkus O., Boekhorst J., Goffau M.D., Smid E.J., Zoetendal E.G., Kleerebezem M. (2013). Diversity of human small intestinal Streptococcus and Veillonella populations. FEMS Microbiol. Ecol..

[41] Backhed F., Ley R.E., Sonnenburg J.L., Peterson D.A., Gordon J.I. (2015). Host-bacterial mutualism in the human intestine. Science.

[42] O’Toole P.W., Jeffery I.B. (2015). Gut microbiota and aging. Science.

[43] Macfarlane G.T., Macfarlane S. (2017). Bacterial metabolism and health-related bioactive compounds. J. Appl. Microbiol..

[44] Miller T.L., Wolin M.J. (2015). The role of Streptococcus in the digestion of dietary fibers. J. Appl. Microbiol..

[45] Slavin J.L. (2013). Dietary fiber and body weight. Nutrition.

[46] O’Leary K. (2021). Breastfeeding supports healthy gut bacteria in infants. Nat. Med..

[47] Backhed F., Roswall J., Peng Y., Feng Q., Jia H., Kovatcheva-Datchary P., Li Y., Xia Y., Xie H., Zhong H. (2015). Dynamics and stabilization of the human gut microbiome during the first year of life. Cell Host Microbe.

[48] Bokulich N.A., Chung J., Battaglia T., Henderson N., Jay M., Li H., D Lieberman T.D., Wu F., Perez-Perez G.I., Chen Y. (2016). Antibiotics, birth mode, and diet shape microbiome maturation during early life. Sci. Transl. Med..

[49] Koenig J.E., Spor A., Scalfone N., Fricker A.D., Stombaugh J., Knight R., Angenent L.T., Ley R.E. (2011). Succession of microbial consortia in the developing infant gut microbiome. Proc. Natl. Acad. Sci. USA.

[50] Manor O., Levy R., Borenstein E. (2016). Mapping the inner workings of the microbiome: Genomic tools for the functional characterization of the microbiota. Cell Host Microbe.

[51] Laursen M.F., Andersen L.B., Michaelsen K.F., Mølgaard C., Trolle E., Bahl M.I., Licht T.R. (2016). Infant gut microbiota development is driven by transition to family foods independent of maternal obesity. mSphere.

